# Phenotypical and Genotypical Properties of an *Arcanobacterium pluranimalium* Strain Isolated from a Juvenile Giraffe (*Giraffa camelopardalis reticulata*)

**DOI:** 10.1155/2014/408724

**Published:** 2014-04-30

**Authors:** Karin Risse, Karen Schlez, Tobias Eisenberg, Christina Geiger, Anna Balbutskaya, Osama Sammra, Christoph Lämmler, Amir Abdulmawjood

**Affiliations:** ^1^Landesbetrieb Hessisches Landeslabor, Schubertstraße 60, 35392 Gießen, Germany; ^2^Frankfurt Zoo, Bernhard-Grzimek-Allee 1, 60316 Frankfurt, Germany; ^3^Institut für Pharmakologie und Toxikologie, Justus-Liebig-Universität Gießen, Schubertstraße 81, 35392 Gießen, Germany; ^4^Institut für Lebensmittelqualität und-sicherheit, Stiftung Tierärztliche Hochschule Hannover, Bischofsholer Damm 15, 30173 Hannover, Germany

## Abstract

The present study was designed to characterize phenotypically and genotypically an *Arcanobacterium pluranimalium* strain (*A. pluranimalium* 4868) following necropsy from a juvenile giraffe. The species identity could be confirmed by phenotypical investigations and by MALDI-TOF MS analysis, by sequencing the 16S rDNA, pluranimaliumlysin encoding gene *pla*, and glyceraldehyde-3-phosphate dehydrogenase encoding gene *gap* with sequence similarities to *A. pluranimalium* reference strain DSM 13483^T^ of 99.2%, 89.9%, and 99.1%, respectively. To our knowledge, the present study is the first phenotypic and genotypic characterization of an *A. pluranimalium* strain isolated from a giraffe.

## 1. Introduction

Genus* Arcanobacterium* was described by Collins et al. 1982 [1] as a group of facultative anaerobic, asporogenous, and Gram-stain positive rods. According to Yassin et al. (2011) [2], this genus consists of four species, namely,* Arcanobacterium haemolyticum*,* Arcanobacterium hippocoleae*,* Arcanobacterium phocae,* and* Arcanobacterium pluranimalium*. More recently,* Arcanobacterium canis* and* Arcanobacterium phocisimile*, two species which were most closely related to* A. haemolyticum*, were described as novel species of this genus [3, 4].

The original species characterization of* A. pluranimalium* was performed with two strains isolated from a dead harbour porpoise and a dead fallow deer [5]. In the following years* A. pluranimalium* could also be isolated from a dog with pyoderma [6], from ovine specimens on 33 occasions, and from a milk sample of a single cow with mastitis [7]. More recently several* A. pluranimalium *strains recovered from various specimens were identified phenotypically and by using various molecular targets [8].

## 2. Material and Methods

The present study was focused on the characterization of an* A. pluranimalium *strain following necropsy from a juvenile giraffe by various phenotypic properties, by MALDI-TOF MS analysis, and genotypically by sequencing 16S rDNA and the* A. pluranimalium-*specific target genes* pla* and* gap*.

The 80.5 kg female giraffe (*Giraffa camelopardalis reticulata*) of the present study was born in 2013. The giraffe was not accepted by its mother or wet nurse and did not accept hand rearing attempts and, because of general weakness, was euthanized three days after birth. The subsequent postmortem analysis revealed an acute hyperemia of lung and liver and a focal emphysema of the lung. The acute pneumonia was caused by a bacterial infection associated with aspirated foreign bodies.

Bacteriological investigations yielded the isolation of* A. pluranimalium *and* Escherichia coli*, partly together with coagulase negative staphylococci, *α*-haemolytic streptococci, and* Pseudomonas fluorescens* from liver, spleen, kidney, and lung. A moderate to high growth of* E. coli *was generally noted (++, +++);* A. pluranimalium *grew only in low numbers (+). The* A. pluranimalium* strain 4868, originally obtained from the spleen, was used for further studies. The bacterial strain was investigated phenotypically and by MALDI-TOF analysis [6, 9] and genotypically by amplification and sequencing of 16S rDNA using universal oligonucleotide primer 16 UNI-L (5′-AGA-GTT-TGA-TCA-TGG-CTC-AG-3) and 16 UNI-R (5′-GTG-TGA-CGG-GCG-GTG-TGT-AC-3) for amplification, under the following PCR conditions: (×1 (95°C, 600 sec), ×30 (95°C, 30 sec, 58°C, 60 sec, 72°C, 60 sec), and using oligonucleotide primer 533-F (5′-GTG-CCA-GCM-GCC-GCG-GTA-A′-3) and 907R (5′-CCG-TCA-ATT-CMT-TTG-AGT-TT-3′) for sequencing. The strain was also characterized by amplification of the target gene* pla* with the oligonucleotide primer* pla*-F: 5′-GTT GAT CTA CCA GGA TTG ACG C-3′ and* pla*-R: 5′-TTG TCG GGG TGT CCA TTG CC-3′ and gene* gap* with the oligonucleotide primer* gap*-F 5′-TTG ACC GAC AAC AAG ACC CT-3′ and* gap*-R 5′-CCA TTC GTT GTC GTA CCA AG-3′as described [8, 10]. Alignment studies were performed using DNASTAR Lasergene Version 8.0.2 (DNASTAR Inc., Madison, WI, USA), Clustal W method. For MALDI-TOF MS the isolates were prepared using the direct smear method as well as an extraction protocol provided by the manufacturer. Briefly, freshly grown bacteria were harvested and diluted in ethanol, centrifuged (2000 ×g), air-dried, and resuspended in aqueous volumes of 70% formic acid and acetonitril followed by a vortex step. Five microliters was directly transferred to the steel target. Analysis was performed on a MALDI-TOF MS Biotyper Version V3.3.1.0. The database used (DB 4613, Bruker Daltonics) comprised 45 spectra from* A. pluranimalium *DSM 13483^T^.

## 3. Results and Discussions

*A. pluranimalium *4868 investigated in the present study was identified by determination of hemolysis and CAMP-like hemolytic reactions, by using a commercial identification system as well as various other phenotypical tests. The CAMP-like hemolytic reactions with* Staphylococcus aureusβ*-hemolysin,* Rhodococcus equi,* and* Arcanobacterium haemolyticum* as indicator strains are known as typical characteristics of this species [6, 8, 11]. Comparable to previously investigated* A. pluranimalium* [6, 8] the phenotypical tests also revealed the typical biochemical properties of this species (Table 1). It was of interest that* A. pluranimalium* 4868 of the present study was catalase negative. This was observed previously for* A. pluranimalium* of bovine origin [8].

As shown by numerous authors MALDI-TOF MS is a powerful tool for species identification of a broad spectrum of bacteria including Gram-positive and Gram-negative bacteria [12–14]. Comparable to the previously conducted MALDI-TOF MS analysis of bacteria of genera* Arcanobacterium* and* Trueperella* (formerly belonging to genus* Arcanobacterium* [9, 15]), MALDI-TOF MS allowed the identification of* A. pluranimalium* 4868 of the present study to the species level matching to* A. pluranimalium *reference strain DSM 13483^T^ with a log score value of 2.28.

**Figure 1:**
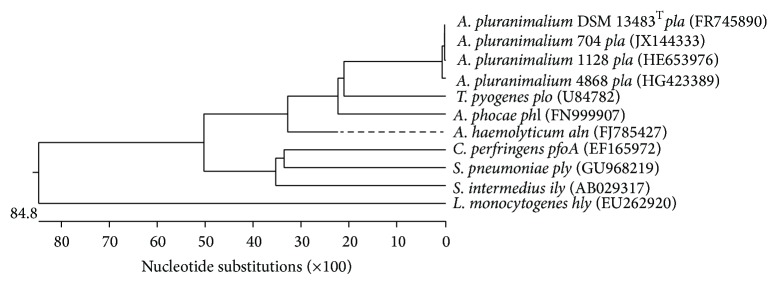
Dendrogram of sequences of gene* pla* of* A. pluranimalium* 4868 of the present study, three additional* A. pluranimalium,* and various other cytolytic toxin encoding genes obtained from GenBank.

**Figure 2:**
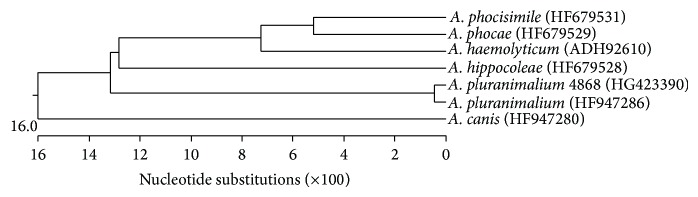
Dendrogram of gene* gap* of* A. pluranimalium* 4868, reference strain* A. pluranimalium* DSM 13483^T^, and various other species of genus* Arcanobacterium *obtained from GenBank.

Sequencing 16S rDNA, the potentially cytolytic toxin pluranimaliumlysin encoding target gene* pla* and the glyceraldehyde-3-phosphate dehydrogenase encoding target gene* gap* revealed a sequence similarity of 99.2%, 89.9%, and 99.1% to the respective sequences of* A. pluranimalium *DSM 13483^T^. All three sequences of* A. pluranimalium* 4868 were deposited in GenBank (HG794511, HG423389, and HG423390). A typical dendrogram of the sequencing results of the genes* pla* and* gap* is shown in Figures 1 and 2. Comparable to gene* plo* of* T*.* pyogenes*, which appeared to be a constant characteristic of all investigated* T*.* pyogenes* [16–19],* pla* of* A. pluranimalium* seems to be also constantly present in all strains of this species and could be used, as described previously [8], and in the present study for molecular identification of* A. pluranimalium*. More recently, Moser et al. 2013 [20] also described* pla* as novel target for molecular identification of this species.

Sequencing of gene* gap* had already been described for molecular identification of staphylococcal species [21] and more recently for identification of an* A. haemolyticum* strain isolated from a donkey [10]. In the present study gene* gap* could also be used as novel target for identification of* A. pluranimalium*. Further studies will give information about the constant presence and sequence similarities of both target genes* pla* and* gap*, respectively.

## 4. Conclusion

The clinical importance of* A. pluranimalium *of the present study, which was isolated from various organs of the giraffe together with in high number appearing* E. coli*, remains unclear. Since, beside aspiration pneumonia, no other pathological findings could be detected, this might represent the route of infection. However, the isolation of this bacterial species from giraffe and the hitherto described origin harbor porpoise, fallow deer, dog, sheep, and cow emphasizes the species name* A. pluranimalium*.

## Figures and Tables

**Table 1:** Biochemical properties of *A. pluranimalium* 4868 investigated in the present study and *A. pluranimalium *DSM 13483^T^.

Biochemical properties	*A. pluranimalium* 4868	*A. pluranimalium* DSM 13483^T∗∗^
Hemolysis on sheep blood agar	+	+
CAMP-like reaction with:∗		
*Staphylococcus aureus* *β*-hemolysin	+	+
*Streptococcus agalactiae *	−	−
*Rhodococcus equi *	+	+
*Arcanobacterium haemolyticum *	+	+
Reverse CAMP reaction	−	−
Nitrate reduction	−^1^	−^1^
Pyrazinamidase	+^1^	+^1^
Pyrrolidonyl arylamidase	+^1^	+^1,2^
Alkaline phosphatase	−^1^	−^1,2^
*β*-Glucuronidase (*β*-GUR)	+^1,2,3^	+^1,2,3^
*β*-Galactosidase (*β*-GAL)	−^1^, (+)^3^	−^1^, (+)^3^
*α*-Glucosidase (*α*-GLU)	−^1,2,3^	−^1,2,3^
*β*-Glucosidase (*β*-GLU)	+^2^	+^2^
N-Acetyl-*β*-glucosaminidase (*β*-NAG)	−^1,3^	−^1,3^
Esculin (*β*-glucosidase)	(+)^1^	+^1^
Urease	−^1^	−^1^
Gelatine	+^1^	+^1^
Fermentation of:		
Glucose	+^1^	+^1^
Ribose	+^1^	+^1^
Xylose	(+)^1^	−^1^
Mannitol	−^1^	−^1^
Maltose	−^1^	(+)^1^
Lactose	−^1^	−^1^
Saccharose	−^1^	−^1^
Glycogen	−^1^	−^1^
*α*-Mannosidase	−^2^	+^2^
Catalase	−	+

The reactions are shown as follows: ∗synergistic CAMP-like reaction with indicator strains; ∗∗results mostly obtained from Ülbegi-Mohyla et al., 2010 [6]; +: positive reaction; (+): weak positive reaction; −: negative reaction. ^1^Api-Coryne test system (Biomerieux, Nürtingen, Germany); ^2^tablets containing substrates (Rosco Diagnostica A/S, Taastrup, Denmark); ^3^4-methylumbelliferyl conjugated substrates (Sigma, Steinheim, Germany).
